# Implementing a Mobile Health System to Integrate the Treatment of Addiction Into Primary Care: A Hybrid Implementation-Effectiveness Study

**DOI:** 10.2196/jmir.8928

**Published:** 2018-01-30

**Authors:** Andrew Quanbeck, David H Gustafson, Lisa A Marsch, Ming-Yuan Chih, Rachel Kornfield, Fiona McTavish, Roberta Johnson, Randall T Brown, Marie-Louise Mares, Dhavan V Shah

**Affiliations:** ^1^ Department of Family Medicine and Community Health University of Wisconsin - Madison Madison, WI United States; ^2^ Center for Health Enhancement Systems Studies University of Wisconsin - Madison Madison, WI United States; ^3^ Center for Technology and Behavioral Health Dartmouth College Lebanon, NH United States; ^4^ College of Health Sciences University of Kentucky Lexington, KY United States; ^5^ School of Journalism and Mass Communications University of Wisconsin - Madison Madison, WI United States; ^6^ College of Letters and Science University of Wisconsin - Madison Madison, WI United States

**Keywords:** mobile health, mHealth, evidence-based practice, behavioral medicine

## Abstract

**Background:**

Despite the near ubiquity of mobile phones, little research has been conducted on the implementation of mobile health (mHealth) apps to treat patients in primary care. Although primary care clinicians routinely treat chronic conditions such as asthma and diabetes, they rarely treat addiction, a common chronic condition. Instead, addiction is most often treated in the US health care system, if it is treated at all, in a separate behavioral health system. mHealth could help integrate addiction treatment in primary care.

**Objective:**

The objective of this paper was to report the effects of implementing an mHealth system for addiction in primary care on both patients and clinicians.

**Methods:**

In this implementation research trial, an evidence-based mHealth system named Seva was introduced sequentially over 36 months to a maximum of 100 patients with substance use disorders (SUDs) in each of three federally qualified health centers (FQHCs; primary care clinics that serve patients regardless of their ability to pay). This paper reports on patient and clinician outcomes organized according to the Reach, Effectiveness, Adoption, Implementation, and Maintenance (RE-AIM) framework.

**Results:**

The outcomes according to the RE-AIM framework are as follows: Reach—Seva reached 8.31% (268/3226) of appropriate patients. Reach was limited by our ability to pay for phones and data plans for a maximum of 100 patients per clinic. Effectiveness—Patients who were given Seva had significant improvements in their risky drinking days (44% reduction, (0.7-1.25)/1.25, *P*=.04), illicit drug-use days (34% reduction, (2.14-3.22)/3.22, *P*=.01), quality of life, human immunodeficiency virus screening rates, and number of hospitalizations. Through Seva, patients also provided peer support to one another in ways that are novel in primary care settings. Adoption—Patients sustained high levels of Seva use—between 53% and 60% of the patients at the 3 sites accessed Seva during the last week of the 12-month implementation period. Among clinicians, use of the technology was less robust than use by patients, with only a handful of clinicians using Seva in each clinic and behavioral health providers making most referrals to Seva in 2 of the 3 clinics. Implementation—At 2 sites, implementation plans were realized successfully; they were delayed in the third. Maintenance—Use of Seva dropped when grant funding stopped paying for the mobile phones and data plans. Two of the 3 clinics wanted to maintain the use of Seva, but they struggled to find funding to support this.

**Conclusions:**

Implementing an mHealth system can improve care among primary care patients with SUDs, and patients using the system can support one another in their recovery. Among clinicians, however, implementation requires figuring out how information from the mHealth system will be used and making mHealth data available in the electronic health (eHealth) record. In addition, paying for an mHealth system remains a challenge.

## Introduction

### The Use of mHealth in Primary Care

Despite the near ubiquity of mobile phones, little systematic research has been conducted on the use or implementation of mobile health (mHealth) technology in managing chronic health conditions in primary care. Although primary care patients use mHealth apps, their use is generally haphazard and self-selected [1,2]. The mHealth apps available to the public vary greatly in quality, and problems such as software bugs, poor design, and limited technical support are common [3]. Perhaps not surprisingly, given these problems, the majority of health-related apps are used only once [4]. Primary care clinicians know very little about the mHealth systems their patients are using, and most clinicians receive no health-related information from mHealth systems [1,5]. The literature contains numerous pilot studies and descriptions of mHealth systems [6] but only a few rigorous studies about the use of mHealth in primary care [5,7]. Implementation research studies in which primary care clinics enroll cohorts of patients using the same mHealth system in an orchestrated fashion are particularly lacking. Thus, the role mHealth can play in the US primary care system remains largely unknown [8].

### mHealth Interventions for Addiction Treatment

Although hundreds of mobile phone apps for addiction treatment are available commercially, most of these apps have not been evaluated in the peer-reviewed literature [9]. The small number of apps that have been evaluated constitute a growing body of evidence supporting the effectiveness of mHealth in treating addiction [10-13]. Most of this evidence relates to self-help interventions [10,11,13] and to texting-based monitoring and reminder systems [9,12]. Little evidence relates to comprehensive mHealth systems for addiction, which have the strongest theoretical base and the most long-lasting effects, or to mHealth interventions for addiction integrated into patients’ recovery and health plans [9].

### Barriers to the Integration of Behavioral Health in Primary Care

In this paper, we report the results of an implementation research trial funded by the National Institutes of Health–National Institute on Drug Abuse. The trial aimed to integrate behavioral health treatment into primary care. We focused on one aspect of behavioral health—addiction—that presents considerable barriers to integration, such as the inability to bill for services, mental health stigma, and primary care physicians being ill-prepared to treat behavioral health problems [14,15]. Primary care operates under productivity guidelines that limit the time clinicians can spend with patients, whereas addiction treatment typically involves frequent counseling sessions. Medication is crucial in primary care, but it has a comparatively short history in addiction treatment [16]. Primary care focuses on chronic conditions, such as diabetes and hypertension, and practitioners expect that patients’ adherence to treatment will vary over time. Behaviorial health has only recently begun to view addiction as a chronic condition [16-18]. Lapses have often resulted in discharge from treatment. Primary care treats patients one-on-one, whereas behavioral health often organizes patients into groups for treatment [19]. Financing models and information technology (IT) infrastructure also differ greatly between the two systems of care [17]. Finally, patients with addictions often have elevated anxiety and present frustrating behaviors to providers, such as frequently missing appointments [20].

In this research, our premise was that mHealth could ease the integration of addiction treatment into primary care. We proposed to examine how implementing an evidence-based mHealth system for addiction could be useful to both patients and clinical staff in real-world primary care settings. The mHealth system used in the study is named *Seva,* a Sanskrit word meaning “selfless caring.” Its key components were previously proven effective in carefully controlled patient-level randomized clinical trials [21,22]. In a randomized clinical trial conducted in patients with alcohol use disorder leaving 90-day residential care, the intervention comprising the backbone of Seva reduced risky drinking days by 57% [21] and increased retention in treatment by 77% [23] compared with patients in the control group.

### Purpose of This Study

The study reported here sought answers to 3 broad research questions:

1. How can Seva be implemented in primary care settings efficiently and effectively?
2. To what extent do patients and staff accept and use Seva?
3. How does Seva affect clinical care for patients and staff?

## Methods

### Study Design

Because the study tested both clinical and implementation interventions, it is considered to be a hybrid type 2 effectiveness-implementation study [24]. Details of the study protocol, the theoretical foundations of the implementation strategy, and a description of Seva were published previously [25]. This paper reports quantitative and observational results. Selected qualitative results were reported separately [26].

We made Seva available to up to 100 patients at each of 3 federally qualified health centers (FQHCs) across the United States of America. FQHCs are federally funded primary care clinics that serve mainly low-income patients. As a condition of funding, FQHCs must provide access to behavioral health services. Thus, FQHCs are in the vanguard of clinics in the United States trying to integrate behavioral health into primary care. FQHCs also serve very vulnerable patients—many in poverty and suffering from addiction. At each site, patients were enrolled over a 12-month period. After enrollment, patients had access to Seva for 12 months.

Because the focus of the study was implementation and not patient outcomes *per se*, the study did not randomize patients. Instead, clinicians were free to enroll any patients from their substance-using populations whom they thought might benefit from Seva based on their clinical judgment. We used the Reach, Effectiveness, Adoption, Implementation, and Maintenance (RE-AIM) framework to organize the evaluation [27]. The RE-AIM framework is a predominant evaluation framework in implementation research studies.

### Ethics

The study protocol was approved by the Medical Sciences Institutional Review Board at the University of Wisconsin–Madison (2012-0937-CP020) and is registered at ClinicalTrials.gov (NCT01963234).

### Clinic Recruitment

Clinics were recruited in partnership with the National Association of Community Health Centers. We recruited FQHCs with established electronic health (eHealth) records to understand how Seva relates to existing clinic technology. In selecting sites, we aimed to achieve geographic reach, diversity in patient populations, and differences in organizational structures to better understand how environmental and structural factors might affect implementation. From a pool of approximately 1100 FQHCs nationally, we selected an FQHC affiliated with the University of Wisconsin as a pilot site; a relatively small, rural, freestanding FQHC with integrated behavioral health services (including addiction treatment) as a second site; and an urban FQHC that largely serves a minority population as our third site.

### Clinician and Patient Recruitment

A limited number of clinician subjects consented at each site. These staff subjects worked with the research team to integrate Seva into clinical workflows during the preimplementation phase and subsequently identified, recruited, enrolled, and trained patients to use Seva and monitored patients’ use of the system.

Authorized clinicians identified potential subjects through the electronic health record (EHR). On the basis of their clinical judgment, clinicians were free to enroll patients from their substance-using populations whom they thought might benefit from Seva. The patients had to meet the following inclusion criteria: (1) aged 18 years or older, (2) meet the criteria for substance use disorder (SUD) as per the Severity of Dependence Scale, (3) have no current psychotic disorder severe enough to prevent participation, (4) have no acute medical problem requiring immediate inpatient treatment, (5) are willing to use Seva, and (6) could understand and sign a consent form in English. If a patient was incarcerated during the study, his or her participation was stopped. If he or she was still interested in participating in the study when released from jail, the person was able to rejoin the study. Patient participation was voluntary. Patient subjects were excluded if (1) their condition warranted inpatient detoxification until they were well enough to participate and (2) they were unable to understand and complete the informed consent. Couples were not recruited to the study at the same time to avoid dyadic conflict.

During a clinic appointment, the clinician asked an eligible patient if he or she was interested in learning more about the study. If the patient was interested and gave permission, the clinician notified the site coordinator that a patient was interested in hearing about the project. In the privacy of a clinic exam room or office, the site coordinator explained the study, its benefits, and potential risks of participation. The site coordinator also answered any questions the patient (subject) had. If the patient was interested in participating, he or she was asked to complete the informed consent.

**Figure 1:**
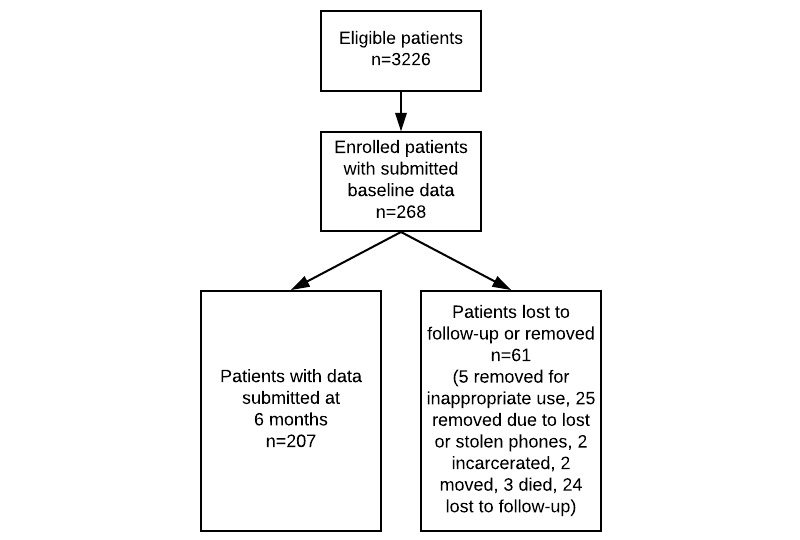
Participant flowchart.

The flow of patients through the study is shown in Figure 1.

### Training Patients on Seva

The site coordinator trained participants in person. Participants could download Seva if they had their own mobile phone. If they did not, the site coordinator gave participants a phone and basic instructions on how to use it. Then he or she explained and showed participants how to use the different services of Seva. Participants were encouraged to ask questions and were given a toll-free number to call the Seva technical support line if they had any questions. Each participant also received a user guide that showed all Seva services and explained how to use them with easy text and graphic instructions.

### Clinic Rollout Process

Each site designated a change leader, a clinical leader who was the point of contact with the implementation coach and coordinated implementation activities; the site coordinator; and a change team of 4 to 8 clinical and/or administrative staff members who helped make the organizational changes that were necessary to implement Seva. The coach made an initial site visit during the 4-month period of implementation preparation to create a welcoming environment for Seva. During this visit, the coach conducted a walk-through exercise with the change team members (an exercise in which employees experience clinic processes as patients do); a workflow assessment using flowcharting; and a technical assessment of data to be gathered and procedures needed to conduct the study. Through this preparation period, the coach worked with the change leader to ensure that pretest data were collected, Seva was demonstrated to clinical teams who might serve as referral sources, and barriers to implementation were identified and rectified.

### mHealth Intervention

Seva offered patients a discussion board used by the patients in the study; interactive modules to teach problem-solving, self-regulation, and other skills; tools for coping with cravings and high-risk situations (eg, relaxation exercises, strategies from cognitive behavioral therapy, links to local 12-step meetings); and health tracking. For clinicians, Seva provided a Web portal with a Clinician Report containing longitudinal information generated by patients’ self-reported data about their substance use and well-being (eg, sleep, depression).

### Implementation Strategy

We created a detailed implementation plan tailored to each clinic. The implementation strategy involved 4 phases at each site, which were (1) initiate (bring key clinical staff together for training), (2) prepare (assure a welcoming environment for Seva), (3) improve (conduct rapid-cycle tests of ideas from the previous stages), and (4) implement (use, monitor, and sustain the technology). An organizational coach (a member of the research team experienced in coaching) helped clinics implement Seva, starting with an in-person planning and kick-off meeting. This meeting was to be followed by monthly phone calls. Implementation plans consisted of a list of scheduled activities, such as when recruitment would start and end, when patients would be trained to use Seva, and so on. The implementation plans were informed by baseline assessments of readiness for implementation, sustainability potential, and technology acceptance from the perspective of clinic staff members who worked on the implementation plan [28-30].

We expected that Seva would enable primary care physicians to better manage addiction in their patients, much as they manage chronic conditions such as diabetes and asthma, by making information about a patient’s recovery readily available. Physicians and other clinicians who referred a patient to behavioral health could see through Seva how these referrals worked out. Seva would also improve outcomes by making support, information, and skills training available to patients almost anywhere and anytime.

### Outcomes

The measures used in this study are summarized in Table 1.

**Table 1:** List of measures.

Domain and measure		Data source(s)
**Reach**		
	Number of Seva patients (eligible, enrolled)	EHR^a^, patient surveys
	Characteristics of participating patients	Patient surveys
**Effectiveness**		
	Substance use	Patient surveys
	QoL^b^	Patient surveys
	Health care utilization (hospitalizations, ER^c^ visits, specialty addiction treatment)	Patient surveys
	HIV testing rates	Patient surveys
	HIV risk behaviors	Patient surveys
**Adoption**		
	Characteristics of participating clinics	Clinic administrative data
	Use of Seva by staff (including referrals)	Seva server files, referral tracking logs maintained by clinic staff
	Use of Seva by patients	Seva server files
**Implementation**		
	Stages of Implementation Completion	Project tracking logs maintained by research team
	Implementation and operating costs	Observation and interviews of clinic staff; project administrative data
**Maintenance**		
	6-month follow-up on effectiveness measures	Patient surveys
	Patient use of Seva at 12 months	Seva server files

^a^EHR: electronic health record.

^b^QoL: quality of life.

^c^ER: emergency room.

To assess *Reach*, we examined the characteristics of the participating sites and the patients they serve. We examined how many patients were eligible to use Seva in each clinic versus how many enrolled in the study over the enrollment period.

To assess *Effectiveness*, we considered patients’ substance use, quality of life (QoL), health care utilization, human immunodeficiency virus (HIV) risk behaviors, and HIV testing rates through self-reported data collected in surveys administered in person at baseline and by phone at 6 months. Risky drinking days were the number of days in the past 30 days on which, within a period of 2 hours, women consumed more than 3 standard drinks and men more than 4 standard drinks, corresponding to the National Institute on Alcohol Abuse and Alcoholism’s definition of binge drinking [31]. Illicit drug use days were defined as the number of days in the past 30 days on which participants used any illicit drug. QoL was measured using the PROMIS Global Health Scale [32].

Effectiveness outcomes are reported for differences between baseline and 6 months. To handle skew distributions, nonparametric related sample tests (Wilcoxon signed rank tests for continuous and ordinal variables and McNemar tests for binary outcomes) were conducted. *P* values (2-tailed) and effect sizes were reported.

We assessed *Adoption* at both the patient and clinic level. For patients, we assessed weekly rates of Seva use, with *use* defined as a patient accessing any part of Seva beyond the home page during a given week in the 12-month period when patients’ phones and service plans were paid for. We defined *clinician use* in two ways—by the number of log-ins by clinicians per week to the website where Seva data were available and by the number of referrals to Seva. We also tracked the total number of Seva patients enrolled and whether medical providers (doctors of medicine [MDs], residents, physician assistants [PAs], nurse practitioners [NPs], and nurses or behavioral health providers) referred patients to Seva. To assess *Implementation*, we used the Stages of Implementation Completion model [33]. The stages of implementation completion are (1) engagement; (2) consideration of feasibility; (3) readiness planning; (4) staff hired and trained; (5) fidelity monitoring processes in place; (6) services and consultation begin; (7) ongoing services, consultation, fidelity monitoring, and feedback; and (8) competency. We planned to assess *Maintenance* in a follow-up phone survey 6 months after each patient’s 12-month intervention period ended, but this plan was abandoned, as discussed below.

## Results

### Reach

We selected an FQHC in Madison, WI, affiliated with the University of Wisconsin-Madison as our pilot implementation site; a relatively small, rural, freestanding FQHC with integrated behavioral health services, including addiction treatment, in Missoula, MT, was our second site; and an urban FQHC in the Bronx, NY, that serves a largely minority population was our third site (Table 2). In all 3 clinics, a total of 3226 patients were deemed clinically appropriate to use Seva in the 12 months corresponding to the implementation period. This number of appropriate patients represents all patients with a SUD diagnostic code in the EHR. With 268 patients enrolled, the intervention reached approximately 8.31% (268/3226) of patients with substance use issues at these 3 clinics. The reach of Seva to the target population was limited by our ability to pay for phones and data plans for a maximum of 100 patients per clinic. The racial and ethnic composition of patients in the study departs somewhat from those of US adults 18 or older estimated by the Substance Abuse and Mental Health Services Administration to meet the Diagnostic and Statistical Manual of Mental Disorders, 4th Edition (DSM-IV) criteria for alcohol dependence and abuse, with study participants being 67.9% (182/268) white versus 55% nationally; 25.0% (67/268) African American/Black versus 16% nationally; 11.6% (31/268) Other versus 10% nationally; and 14.2% (38/268) Hispanic/Latino versus 20% nationally [34]. At least one clinic indicated more patients could have been enrolled.

### Effectiveness

Tables 3 and 4 show changes in patient outcomes reported from baseline to 6 months. Of 268 enrolled patients, 207 (77.2%) were included in this analysis. Effect sizes were calculated per Cohen [35].

In the substance use domain, significant reductions were observed for the number of risky drinking days (the primary outcome in the clinical trial preceding this implementation study), which declined by 44% [(0.7-1.25)/1.25] from baseline to 6 months and illicit drug-use days, which declined by 34% [(2.14-3.22)/3.22]. Two of the three abstinence outcomes also showed significant improvements (any illicit drug use and/or any drink or drug). Significant effects were found for two of the three QoL scores (overall QoL and mental health). Table 3 also shows a significant reduction in hospitalizations and a trend toward fewer emergency room (ER) visits. Table 4 shows an increase in HIV screening rates. Change in the rates of HIV risk behaviors (eg, condom use) and receiving other addiction treatments appeared to be nonsignificant.

Post hoc analyses assessed the relationship between the extent of Seva use and our study outcomes at 6 months. For each outcome, we used linear or logistic regression, controlling for the value of the outcome at baseline. System use was operationalized as (1) the total number of calendar days during the first 6 months on which individuals used Seva (going beyond the main menu), and (2) the total number of Seva pages viewed in the first 6 months (excluding the main menu). These system use measures were natural log transformed to reduce skewness. We found that participants who used Seva on more days in the first 6 months showed a significant increase in alcohol abstinence (*P*=.02), and participants who loaded more Seva pages showed a significant increase in overall abstinence from both alcohol and drugs (*P*=.01), as well as reduced HIV risk behaviors (*P*=.02). We found no significant associations between system use and risky drinking days, illicit drug use days, health care utilization, or QoL.

**Figure 2:**
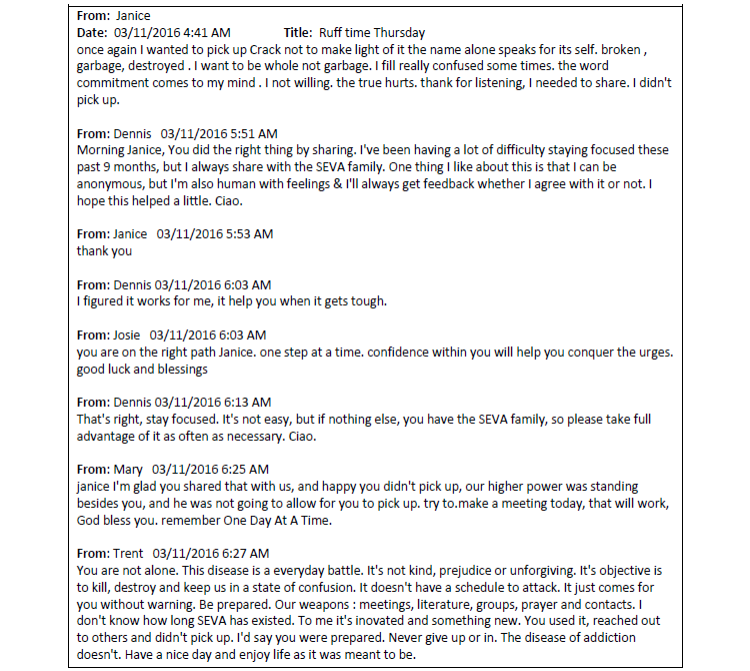
Exchange among Seva patients in the Bronx.

Whereas Seva’s quantitative results show promise in helping primary care patients remain abstinent, other important effects can be best appreciated by directly observing how mHealth affected patient care. An exchange on the Seva discussion board in the Bronx (Site 3) illustrates how the system helped patients support one another. Figure 2 shows an unedited excerpt of this exchange—only names have been changed. The exchange—which occurred over a 2-hour period beginning at 4:41 AM, well before the clinic was open—illustrates how patients struggling with addiction can support one another, in real time, outside the clinic, using mHealth. As the exchange highlights, the network of patients in the Bronx even took to referring to themselves as the “Seva family.”

### Adoption

**Figure 3:**
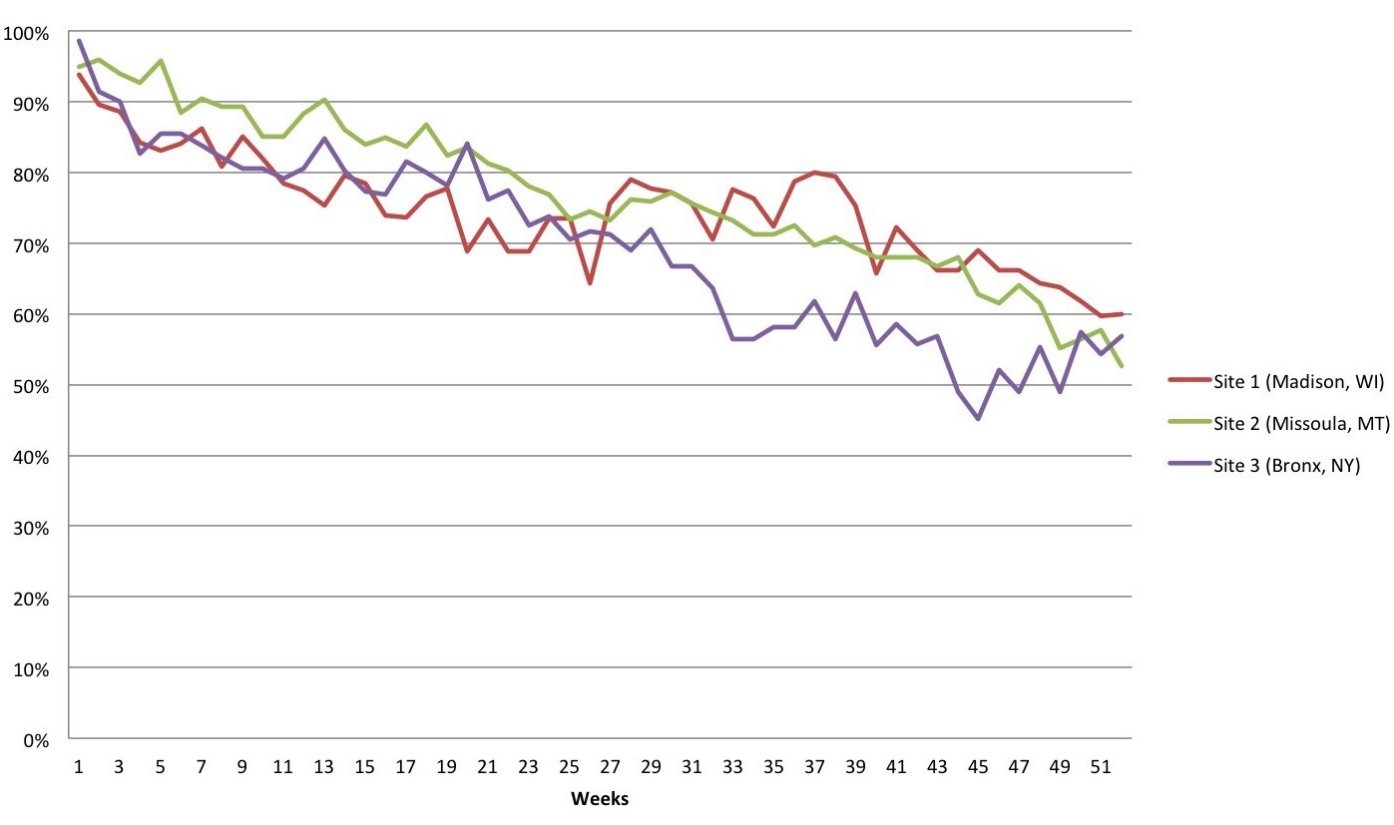
Percentage of patients who logged onto Seva at least once per study week. Patients were excluded from analysis at the point when they were removed from the study (eg, if they lost their phone, died, or were incarcerated).

Mobile health apps generally have low levels of continued use—approximately 80% are abandoned after only 2 weeks [36]. In this context, all 3 sites showed high levels of sustained patient use (Figure 3). At the start of the study, rates of patient use—defined as accessing any part of Seva beyond the home page during each week of the 12-month implementation period—at the 3 sites ranged from 94% (90/96) to 99% (69/70). Rates of use declined, but slowly, with rates at 12 months ranging from 53% (41/78) to 60% (39/65) across the 3 sites, which mirrors the patient retention rate of 57.6% at 8 months in the randomized trial of an earlier iteration of Seva [21].

Clinician adoption of Seva was less robust than patient adoption for two main reasons. At each clinic, clinicians worried about being responsible for data available from Seva. For example, clinicians were concerned that a patient might express suicidal thoughts in a discussion post and they would miss it. To address this concern, the clinical staff at each clinic wanted one staff member to lead the implementation and operation of Seva for the clinic. This job included monitoring patient Seva activity for the clinic and alerting clinicians of significant changes in patient status. At 2 of the 3 sites, members of the behavioral health department led the implementation and operation of Seva. At the other site, the director of innovation led implementation. Not all FQHCs have such a position. At one of the sites, the behavioral health provider who led the implementation of Seva left the clinic near the end of the patient enrollment period. Members of the research team increased their involvement at the site to pick up the slack, but staff turnover remained a vexing issue.

**Table 2:** Baseline characteristics of participating clinics and patients.

Characteristics				Site 1 (Madison, WI)	Site 2 (Missoula, MT)	Site 3 (Bronx, NY)
**Clinic characteristics**						
	**Insurance of patients (%)**					
		**Insured**				
			Medicare	8.7	11.6	11.1
			Medicaid	56.3	23.1	46.5
			Private/other	18.5	19.4	28.9
		Not insured		16.5	45.9	13.5
	eHealth^a^ records			Epic	eClinicalworks	Epic
	Services offered			Primary care and mental health	Primary care, mental health, and addiction	Primary care and mental health
	PCMH^b^ designation			Level 3 (2011)	Level 3 (2014)	Level 3 (2014)
**Patient characteristics**						
	Number of eligible SUD^c^ patients			1189	961	1076
	Patients enrolled in Seva			97	100	71
	**Age (years)**					
		Range		21-64	21-66	22-64
		Mean (standard deviation)		41.61 (10.95)	42.53 (10.24)	42.66 (11.78)
	**Gender n, (%)**					
		Female		52 (54)	40 (40)	36 (51)
	**Drug of choice, n (%)**					
		Alcohol		34 (35)	44 (44)	27 (38)
		Opiates		31 (32)	14 (14)	8 (11)
		Crack cocaine		9 (9)	3 (3)	11 (16)
		Marijuana		1 (1)	4 (4)	16 (23)
		Methamphetamine		0 (0)	15 (15)	1 (1)
		Multiple drugs		22 (23)	20 (20)	8 (11)
	**Ethnicity n (%)**					
		Hispanic/Latino		1 (1)	2 (2)	35 (49)
	**Race, n (%)**^d^					
		White		68 (70)	90 (90)	24 (33)
		African American/Black		30 (31)	2 (2)	35 (50)
		American Indian or Alaskan Native		4 (4)	8 (8)	1 (2)
		Asian or Pacific Islander		0 (0)	1 (1)	0 (0)
		Other		0 (0)	2 (2)	15 (21)

^a^eHealth: electronic health.

^b^PCMH: patient-centered medical home. Three levels of recognition exist, based on practice sites meeting six standards. Level 3 clinics have the best adherence to the standards.

^c^SUD: substance use disorder.

^d^Percentages do not add to 100 because patients could select more than one race.

**Table 3:** Effectiveness results, continuous patient outcomes

Measures		Sample size	Baseline	6 months	Z^a,b^ (*P* value)	Effect size *d*^b,c^
		N	Mean (SD)	Mean (SD)		
**Substance use in last 30 days**						
	Any drinking days	207	2.53 (6.01)	1.67 (4.69)	-2.304 (.02)	-0.228
	Risky drinking days^d^	207	1.25 (3.78)	0.70 (2.58)	-2.008 (.4)	-0.199
	Illicit drug-use days	206	3.22 (7.57)	2.14 (6.55)	-2.499 (.01)	-0.248
**QoL**^e^						
	Overall QoL	202	28.47 (6.46)	30.03 (7.11)	3.653 (<.001)	0.370
	Physical subscale QoL	206	13.20 (3.01)	13.48 (3.11)	1.682 (.09)	0.167
	Mental subscale QoL	204	9.75 (2.99)	10.77 (3.50)	3.892 (<.001)	0.393
**Health care utilization in last 6 months**						
	No. of hospitalizations^d^	207	0.43 (1.03)	0.22 (0.65)	-3.357 (.001)	-0.335
	No. of ER^f^ visits^d^	207	1.10 (2.79)	0.75 (1.31)	-1.911 (.06)	-0.189

^a^Z, provided in the Wilcoxon sign test, is the standard normal distributed *Z*-value used to test the significance between outcomes reported at two time points (eg, pretest vs 6 months).

^b^For *Z* and *d* values, negatives mean decreases and positives mean increases in values from baseline to 6 months.

^c^Calculated from effect size *d*. On the basis of Cohen (1988) effect size, small: *d*=0.2, medium: *d*=0.5, large: *d*=0.8.

^d^Risky drinking days, hospitalizations, and ER visits: Those who reported no such events were coded with zero in the number of days of these events.

^e^QoL: quality of life.

^f^ER: emergency room.

**Table 4:** Effectiveness results, dichotomized patient outcomes

Measures		Sample size	Baseline	6 months	Chi-square (*P* value)	Odds ratio^a^
		N	n (%)	n (%)		
**Substance use in last 30 days**						
	Any drink (Yes)	207	64 (30.9)	51 (24.6)	3.2 (.07)	0.552
	Illicit drug use (Yes)	206	63 (30.6)	36 (17.5)	14.38 (<.001)	0.270
	Any drink or drug (Yes)	206	97 (47.1)	69 (33.5)	12.57 (<.001)	0.349
**Health care utilization in last 6 months**						
	Currently receive other addiction treatments (Yes)	207	89 (43)	78 (37.7)	1.639 (.20)	0.694
**HIV in last 6 months**						
	HIV risky behavior (Yes)	207	76 (36.7)	65 (31.4)	1.818 (.18)	0.667
	HIV testing (Yes)	206	81 (39.3)	116 (56.3)	33.03 (<.001)	—^b^

^a^Numbers lower than 1 mean reductions of the events from baseline to 6 months.

^b^The odds ratio for this variable cannot be calculated because patients’ HIV testing status was considered current at 6 months if they had been tested at baseline; that is, there were zero patients considered tested at pretest and not tested at 6 months.

A second unexpected development affected clinician adoption: We never succeeded in incorporating Seva data into the EHR at each clinic. The interoperability of EHRs is a widely recognized problem. In fact, the same system (eg, Epic Systems, developed by Epic Systems Corporation) may function very differently at one clinic versus another. The technical challenge in getting Seva data into the EHR of 3 health systems proved insurmountable in the context of this implementation research project. Instead, clinicians who wanted to review Seva data had to go to a website outside the EHR.

At each site, the leaders of Seva implementation and operation logged into Seva regularly during the 24 months of enrollment and implementation. At Site 1, 2 other staff clinicians besides the clinic Seva leader from behavioral health were most involved with Seva. The 3 clinicians at Site 1—one primary care physician and 2 behavioral health staff members—logged in an average of 0.6, 0.5, and 1.0 days per week. The Seva clinic leaders at Sites 2 and 3 were also the main users of Seva. The director of innovation at Site 2 logged in an average of 1.7 days per week, and a member of the behavioral health group at Site 3 logged in an average of 4.1 days per week. At two sites, behavioral health providers made most referrals of patients to Seva: 92% (89/97) of referrals at Site 1 and 76% (54/71) at Site 3. At Site 2, on the other hand, where we observed especially strong leadership and operational support for integrating behavioral with medical health, medical providers made 92% (92/100) of referrals (see Table 5).

### Implementation

Table 5 also shows the percent of implementation goals and milestones completed. At the first two sites, we largely executed our implementation plan successfully in the preimplementation and implementation stages. At the third site, we struggled during preimplementation, primarily because of delays in getting the institutional review board’s (IRB) approval, which cost 6 additional months. Cost, which was specified in the protocol as another element of implementation, is reported below.

**Table 5:** Adoption and implementation outcomes.

Measures		Site 1 (Madison, WI)	Site 2 (Missoula, MT)	Site 3 (Bronx, NY)
Total number of Seva patients		97	100	71
Total number of primary care clinical staff at site^a^		70	74	27
Number of patients referred to Seva by primary care clinical staff out of total number of Seva patients, n (%)		8 (8)	92 (92)	17 (24)
Total number of behavioral health providers at site^b^		10	8	11
Number of patients referred to Seva by behavioral health providers out of total number of Seva patients, n (%)		89 (92)	8 (8)	54 (76)
**Time to completion of project phases (months)**				
	Preimplementation (12 months planned)	12	12	18
	Implementation (12 months planned)	18	13	16
	Maintenance (12 months planned)	2	4	1
**Number and percentage of implementation goals and milestones completed, n (%)**				
	Preimplementation (% of monthly implementation plan milestones reached)	12 (100)	12 (100)	12 (100)
	Implementation (% of 100 patient enrollment goal at each site)	97 (97)	100 (100)	71 (71)
	Maintenance (% of 12 monthly coaching follow up calls completed)	2 (17)	4 (33)	1 (8)

^a^Clinical staff members were physicians, residents, physician assistants, nurse practitioners, registered nurses, licensed practical nurses, and medical assistants.

^b^Behavioral health providers were licensed medical social workers, licensed mental health counselors, licensed clinical social workers, doctorate-level psychologists, and physicians.

**Table 6:** Cost analysis (US $).

Type of costs	Site 1 (Madison, WI)	Site 2 (Missoula, MT)	Site 3 (Bronx, NY)
Operating costs (patient and clinic)	113,636	117,150	83,177
Implementation costs (clinic)	9948	23,345	28,121
Total costs for clinic	123,584	140,495	111,298
Cost/patient	1274	1405	1568

Our implementation plans and their execution arose from baseline assessments of readiness for implementation, sustainability potential, and technology acceptance by clinic staff members who worked on the implementation plan, as well as the information that researchers gained from directly interacting with clinic staff. The plans for implementation involved using an organizational coach to help clinics through the 4 phases (initiate, prepare, improve, and implement), using an initial in-person visit and then monthly phone calls between the coach and the clinic change team. Although the organizational coach was heavily involved in the first two phases, another member of the research team who was most familiar with operating Seva became the primary contact for the clinics starting with the third phase. This researcher monitored the Seva discussion groups and gave hands-on, practical advice in response to questions from clinicians. Instead of having monthly coaching calls, clinician-researcher contact consisted mostly of short, frequent, ad hoc phone and email communications between the main clinician users and the research team’s site coordinator in response to specific patient issues and technical questions. These were the types of problems clinics wanted help in solving, rather than helping make organizational change, which was the primary type of help offered by the coach. Thus, we adapted our implementation plan at each site to remove monthly coaching calls during the implementation phase and instead focused on the enrollment of patients as our primary implementation goal.

### Maintenance

Maintenance, defined as continued use of Seva after the 12 months during which patient phones and data plans were paid for, was low at all 3 sites. Patients were allowed to keep their phones and could continue accessing Seva by using Wi-Fi or paying for service themselves with a new phone number. Although we did not track whether patients paid for new data plans independently, we did track Seva use after the 12 months of paid use. Use after 12 months declined gradually to zero once the last recruited patient reached the end of the 12-month period of paid phone use. The decline in use limited our ability to collect follow-up patient surveys and led us to abandon our attempts to collect the phone survey we planned to collect 6 months after each patient’s 12-month intervention period to gauge maintenance. The decline in clinician use of Seva mirrored patient decline. That is, as fewer patients used the system, or if only a few later-enrolled patients were using it, clinicians logged in less because Seva had little patient activity for them to see.

Sites did not continue to use Seva after the study for at least two reasons. First, we could not resolve issues related to transitioning from a research study to ongoing use of an mHealth system. In particular, establishing procedures for consenting patients who wanted to use their own phones to access Seva outside the research protocol, and commingling these patients with patients from the research study, proved challenging. Second, the National Institute of Health grant funding ended, and none of the 3 clinics made arrangements to pay for mobile phones and data plans afterwards. Offering Seva only to patients who can cover their own mobile phone costs could have been a condition of eligibility, but this choice would have shifted the cost to patients and restricted access for low-income patients. It may also have reduced patients’ motivation to use the system, because patients reported that receiving a mobile phone was a strong incentive to use Seva.

Without ongoing funding for patients to use the system, use of Seva declined significantly and patients ultimately became unreachable to the research staff (note that patient surveys were administered over the phone, using the number associated with the patient’s mobile phone). This led us to abandon our attempts to collect the phone survey we planned to collect 6 months after each patient’s 12-month intervention period to gauge maintenance.

### Cost

This analysis addresses cost from the perspective of an FQHC clinic administrator—a comprehensive economic analysis of the study will be reported separately. This analysis includes operating costs (eg, mobile phones, data plans, clinician time for monitoring Seva use, information technology [IT] staff time) and the costs of executing the implementation strategy (eg, site visits, coaching calls). Costs were tracked for 36 months across each project phase (preimplementation, implementation, and maintenance). Costs are broken down as follows: system operating costs; implementation costs per clinic; and overall cost per patient and per clinic (see Table 6). All costs are given in US dollars. Operating costs per patient were estimated at US $1185, which covers US $200 for a mobile phone; US $720 for a voice and data plan (US $60/month x 12 months); US $135 in clinical staff time for patient identification, recruitment, and training (based on observation and interviews with staff—this 2-stage process took an average of 1.5 hours per patient x US $90/hour); and US $130 in staff time for monitoring patients’ use of Seva (based on server logs, clinical and research staff spent 0.12 hours per patient per month monitoring patients; 0.12 hours per patient x 12 months x US $90/hour). We conducted interviews with IT staff members to derive estimates of system operating costs of US $8,000/per clinic over 36 months, which covers costs such as technical support for users, server hosting, and software updates to the system. Implementation costs were estimated at US $10,350 per clinic, which includes US $8,100 for coaching time and expenses associated with site visits (3 visits per clinic at US $500/day, including travel costs of US $1200 per visit) and US $2250 for monthly follow up via email and phone (36 hours x US $62.50/hour).

Total cost per clinic averaged approximately US $124,000 across the 3 clinics; cost per patient averaged approximately US $1,400. For comparison, the average cost for an episode of outpatient addiction treatment among 21 addiction treatment programs surveyed in 2008 (the year with the most recent available cost data) was US $2325 [37].

## Discussion

### Principal Findings

To summarize our findings related to the three research questions that were the focus of the study:

RQ1: How can Seva be implemented in primary care settings efficiently and effectively?

The study offers the following 4 lessons about implementing an mHealth system in primary care:

First, plan and have a budget for working extensively with clinic IT staff to integrate mHealth data into the EHR. This very challenging task is essential for integrating mHealth into primary care because it makes the mHealth data part of rather than separate from the data clinicians expect to see as they treat patients. Second, work with clinic staff to figure out how the mHealth system will fit into the clinic’s existing workflow. All 3 clinics in this study chose to appoint one clinician to monitor Seva data and alert fellow clinicians as needed about important changes in a patient’s recovery. Wider and deeper integration would result from each clinician routinely monitoring data from the mHealth system for his or her patients, just as he or she monitors data related to other chronic conditions such as diabetes. This level of involvement may be unrealistic, however, given the time pressures on primary care staff, and assigning routine monitoring to other clinical staff has been done effectively in other studies we have conducted [38,39]. Third, ensure that the questions clinical staff members have as they operate the mHealth system can get rapid responses from the mHealth developers. Most questions clinicians had in this study were day-to-day operational issues (eg, how to enter a new patient into the system) that were ideally addressed in the moment they occurred by quickly calling the research team. Fourth, to assure sustained use, address cost. Two of the 3 clinics wanted to maintain using Seva based on patient and clinician feedback and, at one site, cost savings from reduced hospital admissions and ER visits. This last clinic used the cost savings to make a case to an insurer to pay for Seva, a process still under way at this writing.

RQ2: To what extent do patients and staff accept and use Seva?

Patient use was exceptionally high compared with continued use of most mHealth apps, although patient use declined steeply after funding for the phones and data plans ended. Clinician use was low compared with patient use because, as stated above, clinicians worried about being responsible for data from Seva and they had to view Seva data in a separate website rather than in the EHR. Of the two aspects of integration we examined—the integration of behavioral health into primary care, and the integration of mHealth into the treatment of addiction in primary care—the second was more successful than the first. Although the treatment of patients suffering from addiction and the use of Seva remained mainly the province of behavioral health providers in this study, the integration of mHealth into addiction treatment was successful if judged by the high levels of patient use.

RQ3: How does Seva affect clinical care for patients and staff?

This study showed the potential of patient peer support in encouraging treatment adherence. Patient peer support is unusual in primary care. It is also a type of care that does not add to, and may reduce, clinician burden. Clinicians who used Seva were generally enthusiastic about it, as demonstrated by 2 clinics wanting to continue using the system after grant funding ended, even though these intentions have thus far gone unfulfilled. The number of clinicians involved, though, is too small to warrant generalizations.

### Comparisons With Prior Work

This study—which involved 3 unaffiliated primary care clinics enrolling nearly 300 patients in the same mHealth system—is the most comprehensive implementation research trial focused on the use of mHealth in primary care yet conducted in the US health care system. Implementation research on mHealth has been focused almost entirely on developing countries in Africa, where mHealth is usually used as a replacement for standard health care and operates independently of any health care system [40].

Prior studies focused on providing addiction treatment to primary care patients have also been rare; we found only 2 clinical trials for primary care SUD interventions [41,42]. Neither of these trials used mHealth. Both addressed illicit drug use and prescription drug abuse rather than AUD and neither had an effect compared with the control group.

We observed significant reductions in drinking and substance use among patients using Seva. These are promising reductions compared with other outpatient AUD treatments. For example, Project MATCH showed that psychosocial treatments for alcoholism are not particularly effective [43], although more recent studies of cognitive-behavioral therapy for SUDs have demonstrated efficacy [44]. A Cochrane review of the effectiveness of naltrexone, a commonly administered medication for AUD, found a decreased risk of heavy drinking by 17% compared with the placebo group and a decrease in the number of drinking days of about 4% [45]. 

Granted, this study was an implementation research trial not specifically designed to retest the effectiveness of Seva. A rigorously designed randomized controlled trial would be required to definitively demonstrate the effectiveness of Seva within a substance-using primary care population.

### Limitations

The study has some limitations. (1) The primary outcome, risky drinking days, is a self-reported measure. (2) Selection bias could have affected which patients participated in the study. Clinicians were advised to enroll any eligible patients they thought might benefit from Seva, based on their clinical judgment. It was not possible for us to tell if this led clinicians to favor some patients over others (eg, those with more education). (3) We could not retrieve racial and ethnic data for SUD patients who were eligible to participate in the study but were not enrolled, and we did not collect data about education and socioeconomic status on Seva patient surveys, limiting our understanding of the representativeness of the patient sample. (4) We lost an opportunity to learn about sustainability from the patient’s perspective because we could not reach patients for the survey we planned to collect 6 months after each patient’s 12-month intervention period. (5) The study would be hard to replicate because it reports on a mobile phone app that, like almost all such apps, changes over time rather than remaining stable. For example, since the start of this study, the app has been rebuilt to update it to current accessibility and security standards, add features (My Motivation), improve features (the automatic tailoring of feedback in response to weekly surveys), and add content for opioid addiction.

### Challenges

The current findings suggest that mHealth faces several formidable challenges to widespread implementation. The first is integrating mHealth with EHRs (eg, Epic Systems), described above. Clinicians in this study had to view Seva data by going to a website outside the EHR, requiring already overburdened clinicians to make extra mouse-clicks. In addition, logistical and patient privacy concerns limited our ability to use EHR data for evaluation purposes. For example, we could not obtain participants’ attendance at primary care visits, which we planned to include in the health care utilization analyses. The poor interoperability and accessibility of EHRs are likely to generalize to other researchers and mHealth developers, making research challenging to conduct and limiting the usefulness of mHealth systems for clinicians.

Another key challenge relates to the difficulty of enrolling patients with SUDs. Clinics reported that it often took multiple follow-up calls to get patients into the clinic to get informed consent and conduct training, even though patients were often excited about getting a mobile phone. The effort required to enroll patients might have dampened enthusiasm for enrollment beyond the first 100 patients that the budget provided for.

FQHCs are required by law to provide behavioral health services. We do not know how Seva might function in a primary care clinic without designated behavioral health staff. Although Seva was somewhat integrated into behavioral health, it did not deeply penetrate physicians' treatment of patients, as reported previously about Site 1 [26] and observed in Sites 2 and 3, perhaps reflecting the deep divides between primary care and behavioral health mentioned in the Introduction [15].

Finally, our experience has revealed several fundamental questions about the role of mHealth in primary care: Is the value of patient peer support for behavioral health sufficient to make the costs of embedding an mHealth system such as Seva into a clinic’s operations worthwhile? Might it instead suffice for patients to use mHealth systems on their own, based upon the recommendation of primary care clinicians? If integrating mHealth into clinic systems is deemed worthwhile, who bears the costs? Certain costs, such as those for mobile phones and data plans, could be borne by patients, some of whom already pay for phones and data plans out of pocket. In this study, patient use of Seva and survey follow-up rates dropped significantly when research funding stopped paying for data plans. Yet, these costs account for only about half the total cost of the system, and lower-income patients often have pay-as-you-go data plans that may not work with the data requirements of an mHealth system such as Seva. Volunteer peer mentors could potentially act as monitors, as clinicians and researchers did in this project, thereby reducing the cost to clinics. Indeed, similar volunteer roles are essential to the Alcoholics Anonymous model (eg, “sponsors”).

### Conclusions

mHealth has the potential to transform health care, and given the enormous cost of health care, we need to make effective use of every available resource. In contrast to the seemingly inexorable rise in health care costs over time, the cost of technology tends to decrease in accordance with Moore’s Law, which posits that computing capability roughly doubles every 2 years. Our experience illustrates that mHealth can engage patients suffering from addiction in ways that benefit patients without adding substantial burden on health care providers. Although challenges remain, thoughtful deployment of mHealth could improve the treatment of addiction in primary care and might also improve the treatment of other chronic conditions that have prominent behavioral components (eg, diabetes). In so doing, mHealth could transcend the physically local and professionally controlled systems that characterize the US health care system.

## Abbreviations

EHR: electronic health record
ER: emergency room
FQHC: federally qualified health center
IT: information technology
mHealth: mobile health
QoL: quality of life
RE-AIM: Reach, Effectiveness, Adoption, Implementation, and Maintenance
